# Identification of a coproporphyrinogen-III oxidase gene and its correlation with nacre color in *Hyriopsis cumingii*

**DOI:** 10.1371/journal.pone.0265318

**Published:** 2022-03-21

**Authors:** Xiajun Chen, Jixiang He, Mengying Zhang, Zhiyi Bai, Jiale Li

**Affiliations:** 1 Anhui Province Key Laboratory of Aquaculture & Stock Enhancement, Fishery Institute of Anhui Academy of Agricultural Sciences, Hefei, China; 2 Key Laboratory of Freshwater Aquatic Genetic Resources, Ministry of Agriculture, Shanghai, China; 3 National Demonstration Center for Experimental Fisheries Science Education, Shanghai Ocean University, Shanghai, China; 4 Shanghai Engineering Research Center of Aquaculture, Shanghai Ocean University, Shanghai, China; Northumbria University, UNITED KINGDOM

## Abstract

Pearl color is an important factor influencing pearl value, and is affected by the nacre color of the shell in *Hyriopsis cumingii*. Coproporphyrinogen-III oxidase (CPOX) is a key enzyme in porphyrin synthesis, and porphyrins are involved in color formation in different organisms, including in the nacre color of mussels. In this study, a CPOX gene (*HcCPOX*) was identified from *H*. *cumingii*, and its amino acid sequence was found to contain a coprogen-oxidase domain. *HcCPOX* mRNA was expressed widely in the tissues of white and purple mussels, and the highest expression was found in the gill, followed by the fringe mantle. The expression of *HcCPOX* in all tissues of purple mussels (except in the middle mantle) was higher than that of white mussels. Strong hybridization signals for *HcCPOX* were observed in the dorsal epithelial cells of the outer fold of the mantle. The activity of CPOX in the gill, fringe mantle, and foot of purple mussels was significantly higher than that in white mussels. Moreover, the expression of *HcCPOX* and CPOX activity were decreased in RNA interference experiments. The findings indicate that *HcCPOX* might contributes to nacre color formation in *H*. *cumingii* by being involved in porphyrin synthesis.

## 1. Introduction

*Hyriopsis cumingii* is a freshwater mussel that is widely distributed in China and produces high-quality freshwater pearls [1]. Pearl size, shape, color, and luster influence the value of pearls, with color being the primary factor [2]. Some studies have indicated that the pearl color of host mussels is affected by the nacre color of the donor mussels [3] and, therefore, nacre color has a substantial impact on the value of pearls. The nacre color of a shell is not only influenced by water environmental factors, but also by genetic factors [4]. Studies have reported that melanin and carotenoids are involved in nacre color formation, and related genes (e.g., *HcTyr*, *HcMitf*, *hcApo*) have also been found to play a role in nacre color in *H*. *cumingii* [5–9].

The pearl oyster shells are usually composed of nacre, prismatic layer and periostracum. The nacre is the inner layer of a shell, and its formation occurs as the shell is made, which involves a complex biomineralization process. Briefly, the gill, foot, and mantle epidermis absorb Ca^2+^ and HCO_3_^−^ from the water, which is transported into the mantle through hemolymph or other mechanisms. These compounds are then secreted by epithelial cells between the hemolymph and mantle cavity, and deposited on the shell. However, some specific mechanisms of this process occur remain unclear [10–12].

Porphyrins are common natural pigments in multiple organisms. Different porphyrins show different colors, such as uroporphyrin is red and protoporphyrin is purple [13]. Moreover, they usually exist in organisms in the form of metal chelates, and the different metal ions they bind will result in different colors. For example, manganese porphyrin and magnesium porphyrin are green, while iron porphyrin is red [14]. Based on the organic chromophore theory and the chemical structure characteristics of porphyrins, Hao *et al*. [15] revealed that there are multiple conjugated double bonds in porphyrins, and its structure belongs to a cyclic conjugated system. The π-π conjugated structure of porphyrin will affect its spectral absorption band. When porphyrin is combined with different metal ions, the original conjugated structure of the porphyrin is distorted or deformed due to differences in the size of the metal ions, which cause the absorption band to shift, resulting in color differences.

Porphyrins are important for the color of organisms. In fact, a large number of studies have shown that porphyrins are the key substances that determine the color of bird eggshells, and that different metal porphyrins can lead to different shell colors [16–19]. Mussel shells are similar to eggshells in that they are both comprised of calcium carbonate. Williams *et al*. found that uroporphyrin I and uroporphyrin III are involved in shell color formation in marine snails [20]. Bonnard *et al*. indicated that the shell color is also related to uroporphyrin in *Crassostrea gigas* oyster [21]. Moreover, studies found that the color of freshwater pearls produced by *H*. *cumingii* related to porphyrins [22]. It is widely known that the nacre formation is similar to the pearl formation, so the effect of porphyrins on the nacre color formation is worth studying.

The synthesis of porphyrin in organisms is a complex process in which coproporphyrinogen-III oxidase (CPOX) is a key enzyme. CPOX catalyzes coproporphyrin III to synthesize protoporphyrinogen IX, and then further synthesis protoporphyrin IX under the action of protoporphyrinogen oxidase, and finally combines with metal atoms to synthesize metalloporphyrin. However, the CPOX effect on shell color has only been reported in *Meretrix meretrix* [23]. Therefore, the effect of CPOX on nacre color in *H*. *cumingii* requires further investigation.

In this paper, the full-length cDNA of the *H*. *cumingii* coproporphyrinogen III oxidase gene (*HcCPOX*) was obtained. The expression of *HcCPOX* and the activity of CPOX in different tissues were also determined. The distribution of *HcCPOX* in the mantle was detected using *in situ hybridization*. RNA interference (RNAi) was performed in purple mussels and the changes in *HcCPOX* expression and CPOX activity were evaluated in different tissues. The findings indicated that *HcCPOX* may affect nacre color by affecting the synthesis of porphyrins.

## 2. Materials and methods

### 2.1 Experimental animals

**Fig 1:**
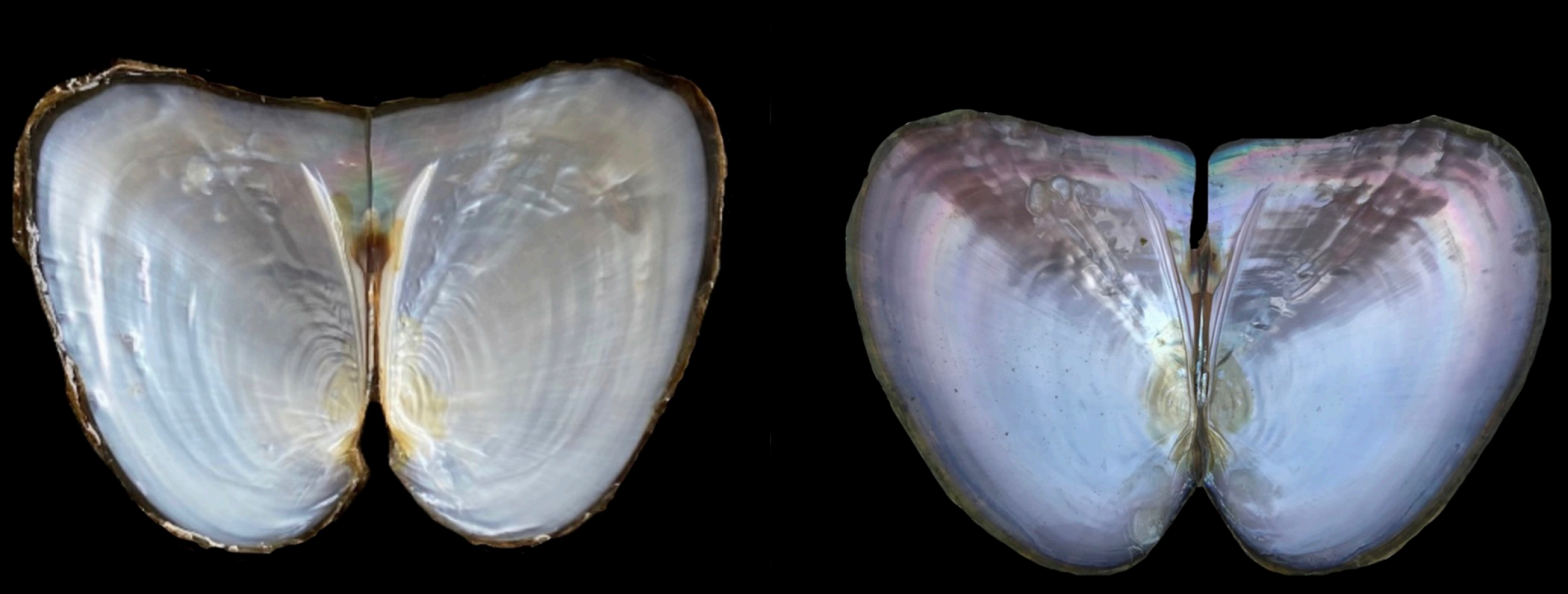
Mussels with white (left) and purple (right) nacre.

Sixty experimental mussels (aged, ~2 years; average shell length, 8.7 cm) were provided by Xuancheng Farm of the Zhexing Pearl Trading Co. Ltd. (Anhui Province, China), including 30 white mussels and 30 purple mussels (Fig 1). Before experimentation, mussels were acclimated for 1 week in tanks containing aerated fresh water (26 ± 1°C).

### 2.2 RNA extraction and full-length cDNA cloning

First, mantle tissue was collected and immediately frozen in liquid nitrogen and stored at −80°C until use. Then, total RNA was extracted using TRIZOL reagent (Invitrogen, Carlsbad, CA, USA), according to the manufacturer’s instructions. The quality of RNA was examined by a spectrophotometer (NanoDrop 2000c, Thermo, USA) and electrophoresis on 1% agarose gels. The SMART RACE cDNA amplification kit (Clontech, Dalian, China) was used to obtain mantle cDNA, and then 5′- and 3′-RACE was performed to clone the full-length cDNA of *HcCPOX*. The specific primers (Table 1) were designed based on the *HcCPOX* expressed sequence tag obtained from the *H*. *cumingii* mantle transcriptome library [24]. The PCR parameters were as follows: 5 min at 94°C; 30 cycles at 94°C for 30 s, 58°C for 30 s, and 72°C for 1 min, and a 10 min final extension at 72°C. The PCR products were sequenced using the Sanger method (Sangon, Shanghai, China).

**Table 1:** Primers used in this study.

Primer name	Sequence (5’-3’)	Application
CPOX-3’	GGGAGTGGCAGCTGTTGAGACGAGGCCG	3’RACE
CPOX-5’	GGACGACAGAGATGTTGACCCC	5’RACE
CPOX-RTF	TATGCAGAAAGGGAGTGGCA	qPCR
CPOX-RTR	TCCCATCTCGCAGTAAGTGG	qPCR
EF1α-F	GGAACTTCCCAGGCAGACTGTGC	qPCR
EF1α-R	TCAAAACGGGCCGCAGAGAAT	qPCR
CPOX-ISHF	TATGCAGAAAGGGAGTGGCA	*In situ* hybridization
CPOX-ISHR	TAATACGACTCACTATAGGGTCCCATCT	*In situ* hybridization
	CGCAGTAAGTGG	
CPOX-iF+T7	GGATCCTAATACGACTCACTATAGG	RNAi
	GTCAACATCTCTGTCGTCCA	
CPOX-iR	CGAGTCTCCTCTCTGTGTTTG	RNAi
CPOX-iF	GTCAACATCTCTGTCGTCCA	RNAi
CPOX-iR+T7	GGATCCTAATACGACTCACTATAGGCGA	RNAi
	GTCTCCTCTCTGTGTTTG	
GFP-iF+T7	GGATCCTAATACGACTCACTATAGGAAG	RNAi
	GGCGAGGAGCTGTTCACCG	
GFP-iR	CAGCAGGACCATGTGATCGCGC	RNAi
GFP-iF	AAGGGCGAGGAGCTGTTCACCG	RNAi
GFP-iR+T7	GGATCCTAATACGACTCACTATAGGCA	RNAi
	GCAGGACCATGTGATCGCGC	

### 2.3 Bioinformatics analysis

The open reading frame (ORF) of *HcCPOX* was predicted using the NCBI ORF Finder tool (http://www.ncbi.nlm.nih.gov/projects/gorf/) [25]. The BLAST program (http://www.ncbi.nlm.gov/blast) was used to perform homology analyses of the nucleotide and protein sequences [25]. The Simple Modular Architecture Research Tool (SMART, (http://smart.embl-heidelberg.de/)) was used to analyze the structural domains contained in the amino acid sequence. The online Protparam (http://www.expasy.org/tools/protparam) tool was used to obtain amino acid sequence composition, molecular weight, isoelectric point, and other physical parameter information [26]. Clustalx software was used to perform multiple sequence comparisons [27]. MEGA 5.2 was used to construct a phylogenetic tree using the maximum likelihood method, and the aligned sequences were bootstrapped till 1000 replicates were obtained [28].

### 2.4 Tissue-specific expression analysis of *HcCPOX*

Quantitative RT-PCR was used to detect the tissue-specific expression of *HcCPOX* in different tissues. Different tissues (fringe mantle, middle mantle, adductor muscle, gill, foot, hepatopancreas, and hemolymph) were obtained from 12 healthy *H*. *cumingii* (six white mussels and six purple mussels), and total RNA extraction was performed as described above. The PrimeScriptTM RT reagent kit with gDNA Eraser (Takara, Dalian, China) was used to synthesize cDNA for qPCR analysis. Specific primers (Table 1) were designed based on the sequence of *HcCPOX*, and EF-1α (Table 1) was used as the internal reference [29]. qPCR was carried out in a quantitative thermal cycler (Bio-Rad CFX-96; Bio-Rad, USA), with the following parameters: 3 min at 95°C; then 40 cycles of 95°C for 5 s, 57.5°C for 30 s and 72°C for 30 s. Finally, tissue-specific expression of *HcCPOX* was analyzed using the comparative CT method (2^−ΔΔCt^) [30].

### 2.5 *In situ* hybridization of *HcCPOX* in the mantle

*In situ* hybridization was used to detect the precise locations of expression of *HcCPOX* in the mantle of purple mussels. First, the RNA probes were synthesized using the T7 *in vitro* Transcription Kit (Promega, USA), according to the manufacturer’s instructions. The mantle samples (including outer fold, middle fold, inner fold of the mantle) were then collected and fixed in 4% paraformaldehyde for 6 h at 4°C, and then placed in 20% sucrose overnight at 4°C [5, 7]. A freezing microtome (Leica CM 1950; Wetzlar, Germany) was used to slice mantle samples into 13-μm thicknesses and stored at −80°C. Finally, *in situ* hybridization was performed according to the manufacturer’s instructions (Enhanced Sensitive ISH Detection Kit, Boster, Switzerland).

### 2.6 Analysis of the coproporphyrinogen-III oxidase activity

Eight mussels (four white mussels and four purple mussels) were used to detect the coproporphyrinogen-III oxidase activity in *H*. *cumingii*. Tissues (gill, fringe mantle, middle mantle, hemolymph) were collected and enzyme activity analyses were performed using the Shellfish Coproporphyrinogen Oxidase ELISA Kit (LanPai, Shanghai, China) according to the manufacturer’s instructions. In brief, 300 mg of each tissue was comminuted in liquid nitrogen. Phosphate-buffered saline (PBS) was then added, centrifuged at 3000 rpm for 10 min at room temperature, and the supernatant was taken as a test sample. Then set standard wells (add 50 μl standard) and testing sample wells (add 10 μl test sample then add 40 μl sample diluent) in the microtiter plate, which pre-coated with CPOX antibody. Horseradish Peroxidase-conjugated reagent (100 μl) was added to each well, covered with an adhesive strip, and incubated for 60 min at 37°C. Each well was then washed with wash solution (400 μl) five times, with complete removal of liquid at each step. Chromogen solution A and B were then added (50 μl) to each well, and incubated for 15 min at 37°C in the dark. Finally, 50 μl of stop solution was added to each well and the O.D. at 450 nm was read using a microplate reader (UV-3010; Shimadzu, Japan). The CPOX activity of *H*. *cumingii* was obtained by comparing the O.D. of the samples with the standard curve.

### 2.7 RNAi assays

Specific primers (Table 1) were designed for double-stranded RNA (dsRNA) synthesis based on the sequence of *HcCPOX*. T7 RNA polymerase (TAATACGACTCACTATAGGG) was added to the 5′ end of the upstream and downstream primers. The PCR product was used for the synthesis of dsRNA using the T7 *in vitro* Transcription Kit (Promega, USA), according to the manufacturer’s instructions. Green fluorescent protein (GFP) dsRNA was synthesized as described above and used as the control group. Twenty-nine healthy purple mussels were divided equally into three groups (experimental group, negative control group, and blank group), and injected with ds*HcCPOX* (80 μg/100 μL), dsGFP (80 μg/100 μL) and 1 × PBS (100 μL) via the adductor muscle. Tissues (gill, fringe mantle, middle mantle, and hemolymph) from each mussel were collected after culturing in freshwater for one week. Finally, the expression of *HcCPOX* and the activity of CPOX in each tissue were detected as described above.

### 2.8 Statistical analysis

Data are shown as the mean ± SD, and a one-way ANOVA was performed using SPSS 17.0 (SPSS, Chicago, IL, USA) to determine whether there were any significant differences. A *p*-value < 0.05 was considered statistically significant.

## 3. Results

### 3.1 Cloning and sequence analysis of *HcCPOX*

**Fig 2:**
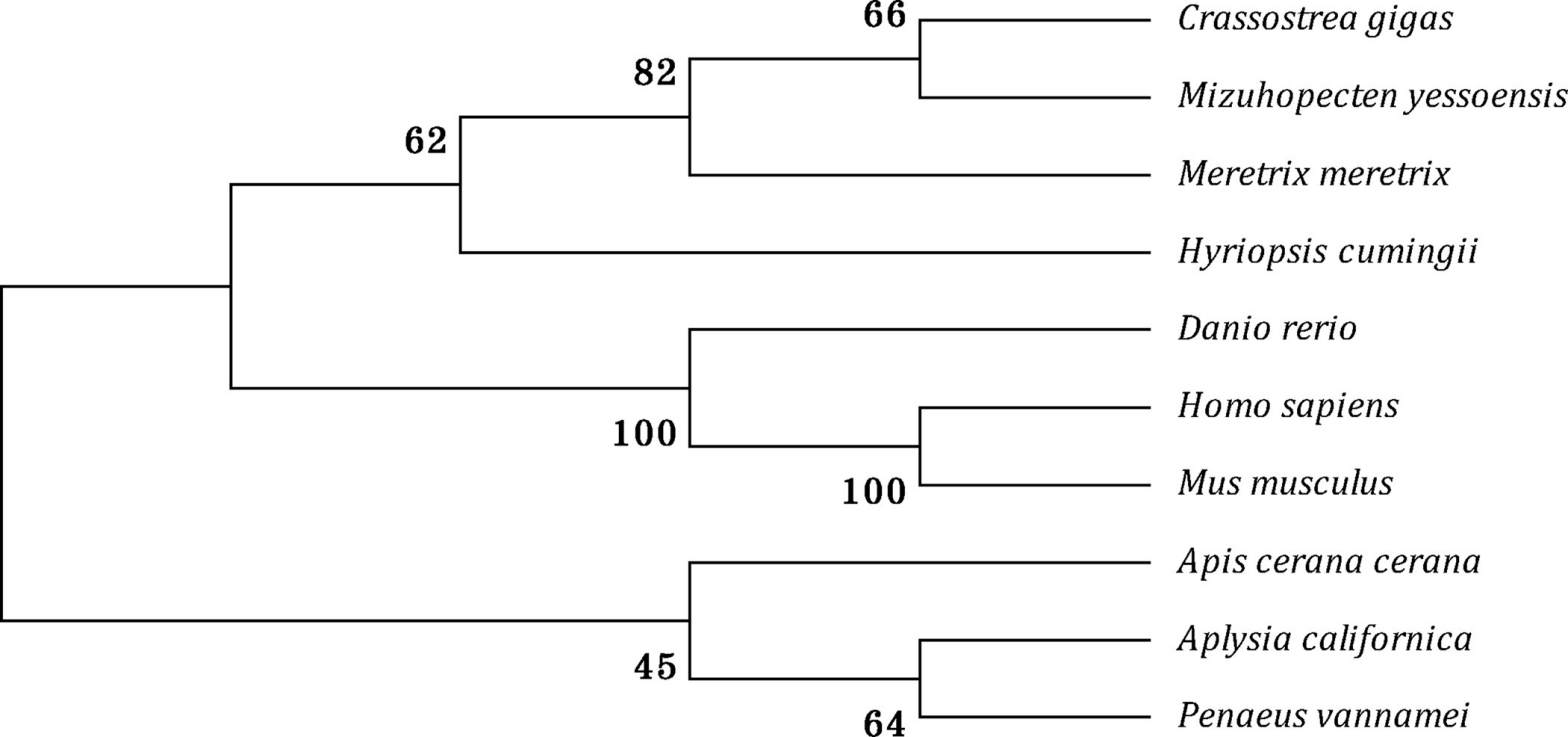
Phylogenetic analysis of *HcCPOX* from *Hyriopsis cumingii* and related species. GenBank accession numbers: *Crassostrea gigas* (EKC32626.1), *Mizuhopecten yessoensis* (OWF48267.1), *Meretrix meretrix* (AMY96566.1), *Hyriopsis cumingii* (KX447817), *Danio rerio* (NP_001035183.2), *Homo sapiens* (NP_005247182.1), *Mus musculus* (NP_031783.2), *Apis cerana cerana* (PBC27150.1), *Aplysia californica* (AAP34327.1), and *Penaeus vannamei* (ROT62892.1). Numbers are the bootstrap values for 1000 trials.

The full-length cDNA of *HcCPOX* (Accession No. KX447817) was obtained by 3’- and 5’-RACE (S1 Fig). It contained a 3-bp 5’-UTR, an 1173-bp open reading frame (ORF) and a 346-bp 3’-UTR. The ORF encoded a polypeptide of 390 amino acids with a calculated molecular weight of 44.9 kDa and a theoretical isoelectric point of 8.26. The ORF also contained a coprogen-oxidase domain (PF01218). A phylogenetic tree was constructed and showed that the amino acid sequence of *HcCPOX* belonged to the same clade as CPOX from *Meretrix meretrix*, *Mizuhopecten yessoensis*, and *Crassostrea gigas*, with a support of 62% (Fig 2).

### 3.2 qPCR analysis and *in situ* hybridization

**Fig 3:**
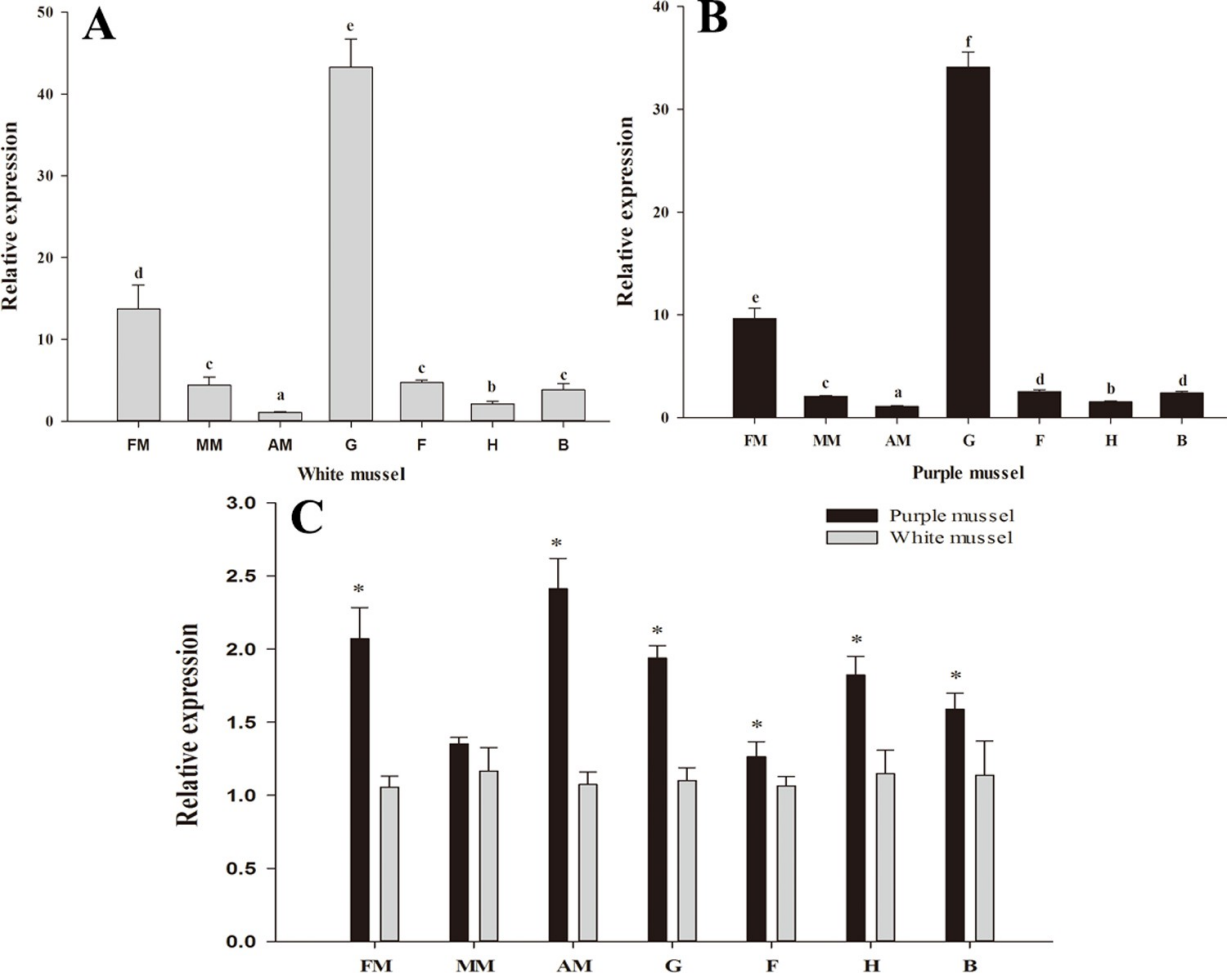
Relative expression of *HcCPOX*. The relative expression of *HcCPOX* in various tissues from white (A) and purple (B) mussels. Comparisons of *HcCPOX* expression in white and purple mussels (C). FM, fringe mantle; MM, middle mantle; AM, adductor muscle; G, gill; F, foot; H, hepatopancreas; B, hemolymph. Bars with different letters or * indicate significant differences (*p* < 0.05), the same as below.

The relative expression level of *HcCPOX* in the tissues of white and purple mussels was detected by qPCR. As shown in Fig 3, *HcCPOX* was expressed in all tissues of white and purple mussels. In white mussels, the tissue with the highest expression was the gill, followed by the fringe mantle. The level of expression in gill and fringe mantle were significantly higher than that in other tissues (*p* < 0.05). No significant differences in the expression levels of *HcCPOX* in hemolymph, foot, and middle mantle were observed (*p* > 0.05). The expression of *HcCPOX* in the adductor muscle was significantly lower than that in the hepatopancreas (*p* < 0.05). In purple mussels, the highest level of *HcCPOX* expression was also in the gill, followed by the fringe mantle. The level of *HcCPOX* expression in the gill and fringe mantle was significantly higher than that in other tissues (*p* < 0.05). No significant differences were observed between *HcCPOX* expression in the foot and the hemolymph (*p* > 0.05). The levels of expression in the remaining tissues followed the order from high to low of middle mantle > hepatopancreas > adductor muscle (*p* < 0.05).

Finally, comparing the expression levels of *HcCPOX* in different tissues of the two types of mussels, it was found that the expression of *HcCPOX* was higher in all tissues from purple mussels (with the exception of the middle mantle) than white mussels (*p* < 0.05).

**Fig 4:**
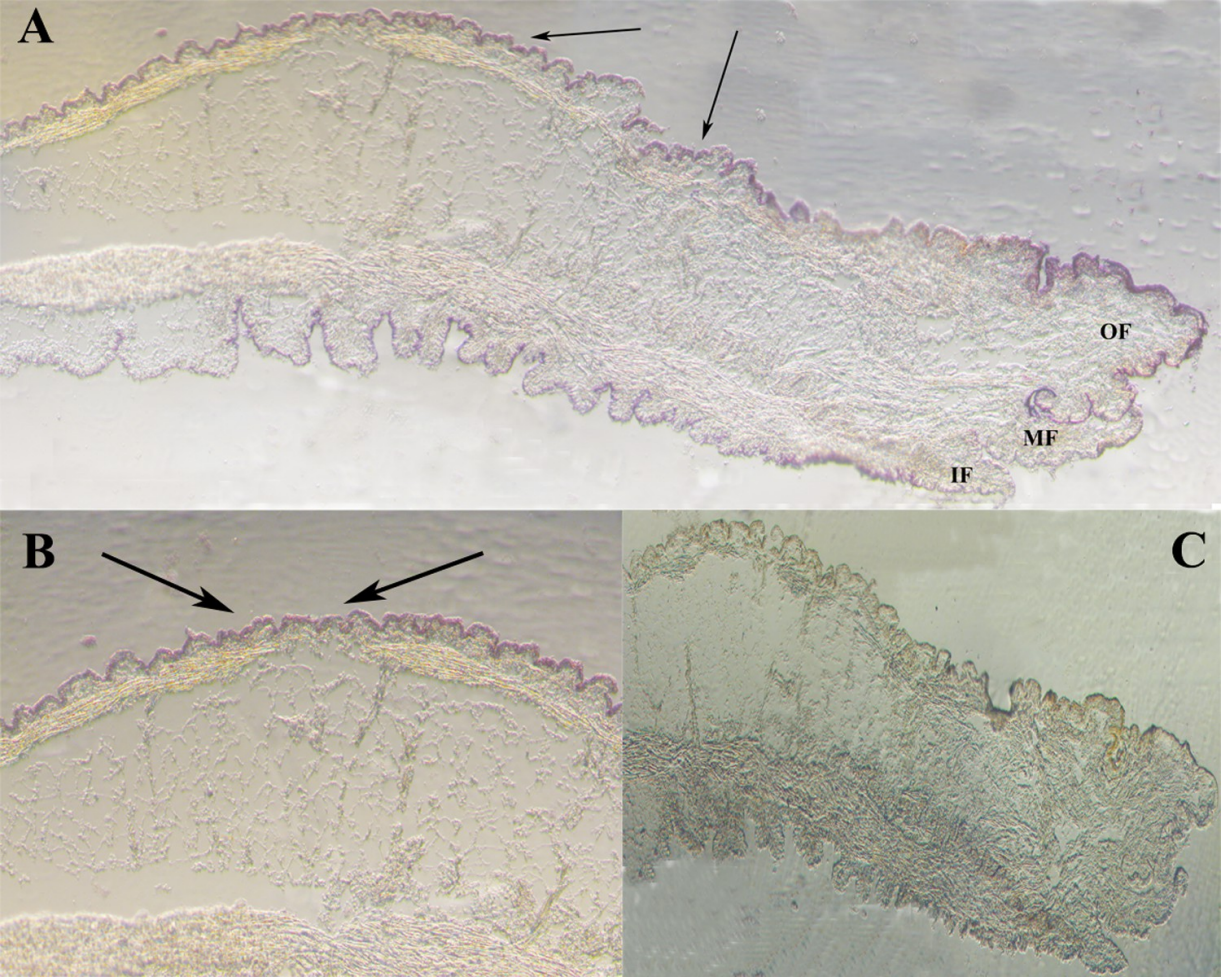
*In situ* hybridization analysis of *HcCPOX* in the mantle. Panel B was a higher magnification of panel A. Panel C represents the negative control (using the sense probe). IF, inner fold; MF, middle fold; OF, outer fold. The arrows indicate the location of the positive signals.

*In situ* hybridization revealed the specific locations of *HcCPOX* expression in the mantle. As shown in Fig 4, strong hybridization signals appeared in the dorsal epithelial cells of the outer fold of the mantle (arrowed), while no positive signals were detected in the negative control.

### 3.3 Coproporphyrinogen-III oxidase activity

**Fig 5:**
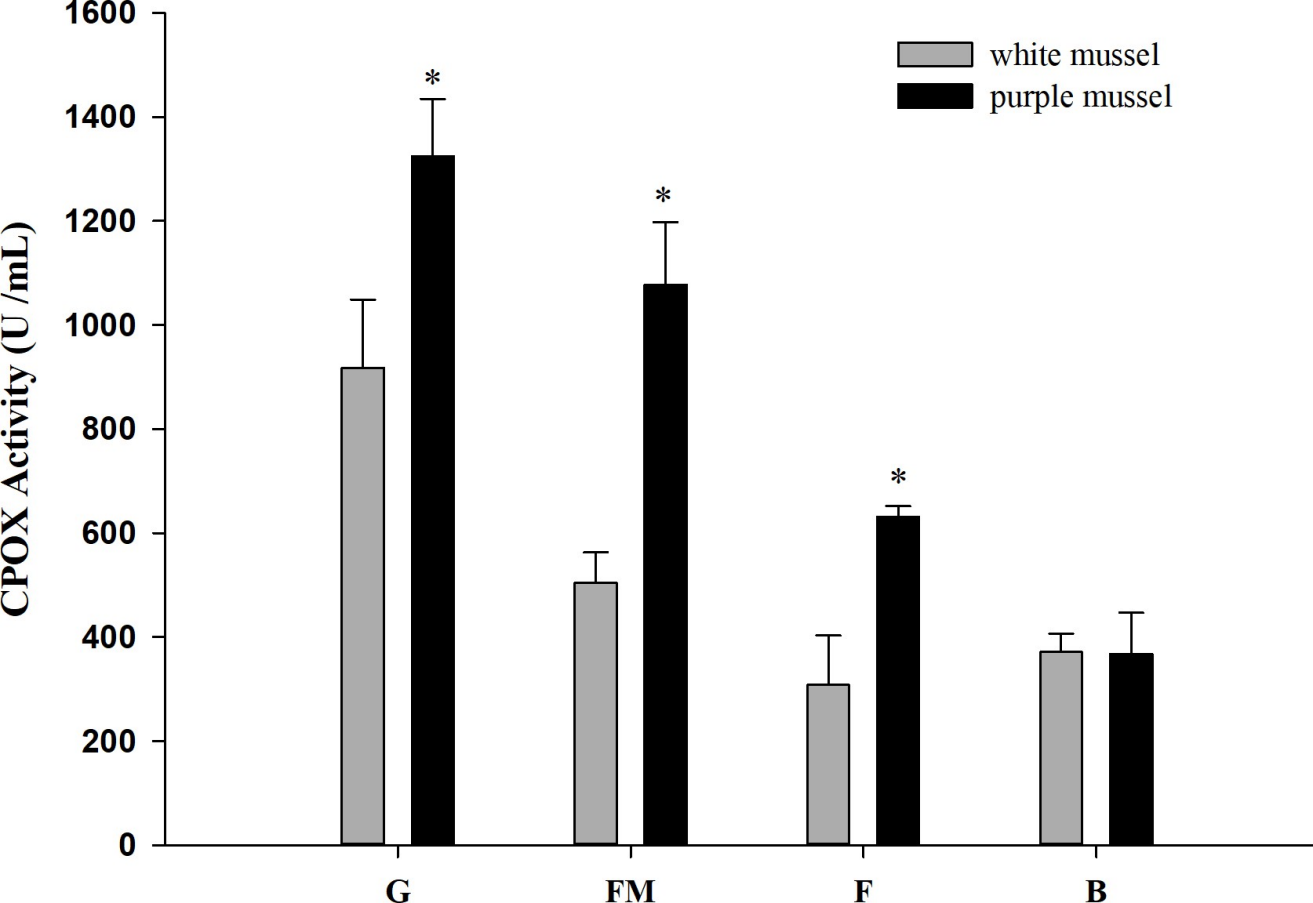
Coproporphyrinogen-III oxidase activity in tissues from white and purple mussels. G, gill; FM, fringe mantle; F, foot; B, hemolymph.

As shown in Fig 5, CPOX activity in mussel tissues was consistent with the *HcCPOX* expression levels. CPOX activity was the highest is the gill, followed by the fringe mantle. Additionally, the activity of CPOX in the gill, fringe mantle, and foot of purple mussels was significantly higher than that of white mussels (*p* < 0.05), however, no significant differences were observed in the hemolymph (*p* > 0.05).

### 3.4 The impact of RNAi

**Fig 6:**
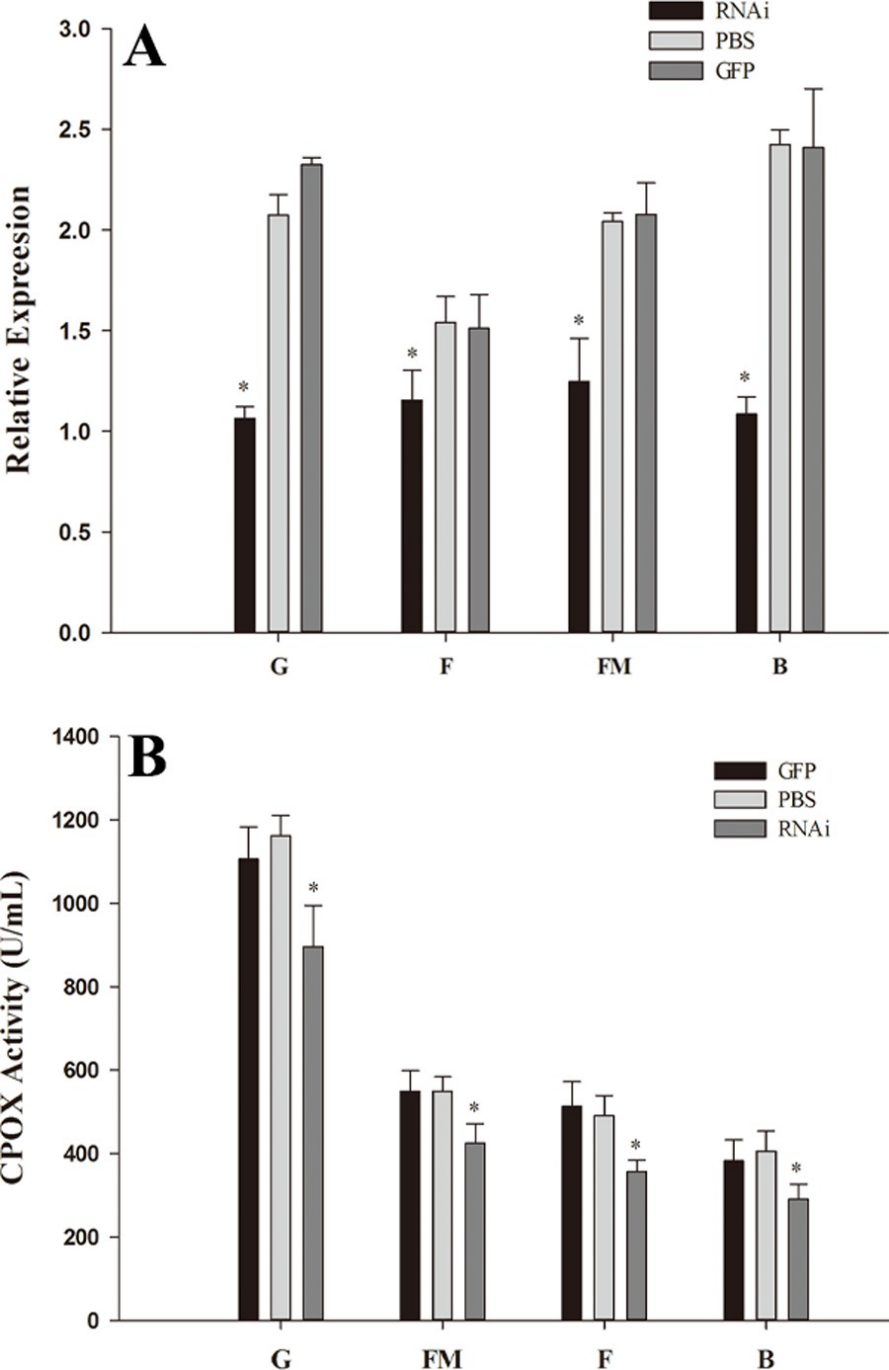
Effects of *HcCPOX* knockdown by RNAi. A, RNAi attenuated HcCPOX expression after RNAi. B, RNAi attenuated coproporphyrinogen-III oxidase activity after RNAi. G, gill; FM, fringe mantle; F, foot; B, hemolymph.

*HcCPOX* expression levels and CPOX activities were detected after RNAi treatment. As shown in Fig 6A, the expression of *HcCPOX* in the gill, fringe mantle, foot, and hemolymph of the mussels injected with ds*HcCPOX* was significantly decreased compared to mussels injected with dsGFP and 1 × PBS (*p* < 0.05). However, the expression of *HcCPOX* in the tissues of mussels injected with dsGFP was not significantly different than that of mussels injected with 1× PBS (*p* > 0.05). Furthermore, CPOX activities in those tissues were also similar to the *HcCPOX* expression levels. After injection of ds*HcCPOX*, CPOX activities in all tissues were inhibited compared to that of mussels injected with dsGFP and 1 × PBS (p < 0.05). Additionally, no significant difference in CPOX activity was observed between mussels injected with dsGFP and 1 × PBS (*p* > 0.05) (Fig 6B).

## 4. Discussion

CPOX is an indispensable enzyme that is required for the synthesis of heme in different organisms. Some studies found that many different pigments (e.g., uroporphyrin, cobalamin, phorcabilin, biliverdin, and bilirubin) are produced in the synthesis and decomposition pathways of heme, which affect the shell color in *Pinctada margaritifera* [31]. If the heme synthesis pathway is affected, it may also lead to the formation of different shell colors in marine snails [20].

In this study, the CPOX gene (*HcCPOX*) from *H*. *cumingii* was successfully cloned. The amino acid sequence contained a coprogen-oxidase domain, which belongs to the conserved sequence of the coproporphyrinogen oxidase superfamily. Phylogenetic analysis also revealed that the *HcCPOX* amino acid sequence was in the same clade as CPOX sequences from shellfish (*M*. *meretrix*, *M*. *yessoensis*, *and C*. *gigas*). Therefore, the data suggested that *HcCPOX* belongs to the CPOX superfamily.

The CPOX gene encodes porphyrinogen III oxidase in organisms, which catalyzes the formation of porphyrin substances via an oxidation reaction. Studies have found that porphyrin plays an important role in the synthesis of haem in the blood of mammals and mollusks [32, 33]. Williams *et al*. identified some genes of the haem pathway in marine snails, which affect shell color by participating in the synthesis of porphyrin [20]. Moreover, similar results were also found in *P*. *margaritifera* [31].

In a study in which the CPOX gene was knocked out in zebrafish, it was found that the hemolymph changed from red to nearly colorless, thus, indicating that CPOX gene is involved in the formation of hemolymph color (heme) in zebrafish [34]. *H*. *cumingii* is a bivalve mollusk, and its hemolymph is typically nearly colorless, which occasionally appears light blue after being fully oxidized. This color change is mainly caused by the small amount of hemocyanin contained within its hemolymph [35].

Combined with the qPCR results, we found that *HcCPOX* was expressed in hemolymph, but only a low levels. This may be due to its colorless hemolymph. In addition, there was no significant difference in CPOX activity in the hemolymph of white and purple mussels, and was much lower than that in the gill and fringe mantle.

The expression of *HcCPOX* in white and purple mussels was highest in the gills. Similar results were also obtained for CPOX activities. Cooper *et al*. found that the gills of mussels were the main locations for the uptake of metal ions from water environments [36]. The high levels of *HcCPOX* expression in the gill may be due to high level of metal ions contained therein, and gill may provide a suitable environment for the synthesis of metalloporphyrins, and then porphyrins transferred to the mantle. Comfort *et al*. indicated that metalloporphyrin is a key substance in the formation of color in shells [37]. Shi *et al*. found that a variety of metal ions exist in the pearls of *H*. *cumingii* by Raman spectroscopy, and speculated that they might affect the shell color in the form of metalloporphyrin [22]. The mantle is the tissue responsible for the nacre formation of shells and pearls. The qPCR results found that the levels of *HcCPOX* in the fringe mantle of white and purple mussels was high, and only lower than that of the gill. Moreover, positive signals were detected in the dorsal epithelial cells of the outer fold of the mantle using *in situ* hybridization. Studies indicated that outer fold of the mantle was directly involved in the nacre formation of shells [38]. Thus, these results suggested that *HcCPOX* may be involved in the formation of nacre in *H*. *cumingii*.

Zhan *et al*. found that the CPOX gene (*MmCPOX*) in *M*. *meretrix* was highly expressed in the mantle, and expression was positively correlated with shell color [23]. The uroporphyrinogen III synthetase (an important enzyme involved in the structural formation of porphyrin) gene was found to be involved in nacre color formation in *H*. *cumingii* [39]. In this study, the expression of *HcCPOX* in tissues of white and purple mussels were compared. The result revealed that the expression levels of *HcCPOX* were significantly higher in all tissues of purple mussels (except the middle mantle) than in white mussels. We also found that the CPOX activity in the tissues (fringe mantle, gill, and foot) of purple mussels were also significantly higher than that in white mussels. The RNAi experiments revealed that the activity CPOX was decreased following the inhibition of *HcCPOX* expression in all tissues. Therefore, we hypothesize that *HcCPOX* might affect nacre color formation by participating in the synthesis of CPOX.

In conclusion, the results of this study indicate that *HcCPOX* contributes to nacre color formation in *H*. *cumingii*. Such findings can help understand the mechanisms underlying the formation of pearl color, and assist in the cultivation of high quality pearls.

## Supporting information

S1 Fig Full-length nucleotide sequence and deduced amino acid sequence of the HcCPOX (Gene accession is KX447817). The gray box indicates the predicted coprogen-oxidase domain.
(DOCX)
