# Supplementary material for: Identification of a coproporphyrinogen-III oxidase gene and its correlation with nacre color in Hyriopsis cumingii
Source: PLoS One. 2022 Mar 21;17(3):e0265318. doi: 10.1371/journal.pone.0265318 (PMC8936452; doi:10.1371/journal.pone.0265318)
Supplement: S1 Fig — The gray box indicates the predicted coprogen-oxidase domain. (DOCX) [file pone.0265318.s001.docx]

**Supplementary**

**Figure S1. Full-length nucleotide sequence and deduced amino acid sequence of the HcCPOX(Gene accession is KX447817). The gray box indicates the predicted coprogen-oxidase domain.**

**Figure S1.**

**
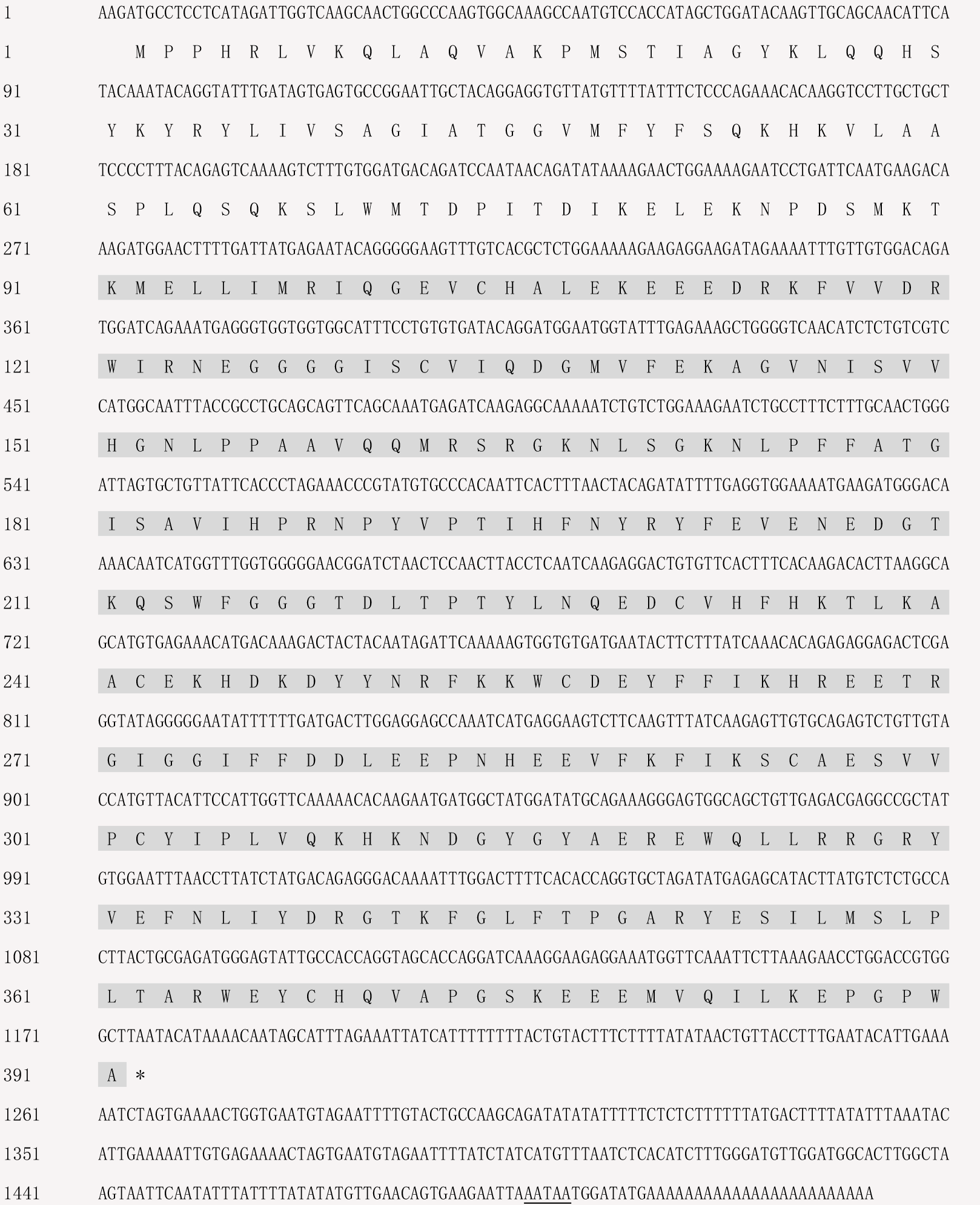
**
